# Sarcopenia: prevalence, associated factors, and the risk of mortality and disability in Japanese older adults

**DOI:** 10.1002/jcsm.12651

**Published:** 2020-11-25

**Authors:** Akihiko Kitamura, Satoshi Seino, Takumi Abe, Yu Nofuji, Yuri Yokoyama, Hidenori Amano, Mariko Nishi, Yu Taniguchi, Miki Narita, Yoshinori Fujiwara, Shoji Shinkai

**Affiliations:** ^1^ Research Team for Social Participation and Community Health Tokyo Metropolitan Institute of Gerontology Tokyo Japan; ^2^ Integrated Research Initiative for Living Well with Dementia Tokyo Metropolitan Institute of Gerontology Itabashi‐ku Tokyo Japan; ^3^ Centre for Urban Transitions Swinburne University of Technology Melbourne Australia; ^4^ Center for Health and Environmental Risk Research National Institute for Environmental Studies Ibaraki Japan

**Keywords:** Community‐based sample, Prospective study, Appendicular lean mass index, Handgrip strength, Gait speed

## Abstract

**Background:**

There is limited evidence on sarcopenia in Asian populations. This study aimed to clarify the prevalence, associated factors, and the magnitude of association with mortality and incident disability for sarcopenia and combinations of its components among Japanese community‐dwelling older adults.

**Methods:**

We conducted a 5.8 year prospective study of 1851 Japanese residents aged 65 years or older (50.5% women; mean age 72.0 ± 5.9) who participated in health check‐ups. Sarcopenia was defined according to the Asian Working Group for Sarcopenia 2019 algorithm. Appendicular lean mass index (ALMI) was measured using direct segmental multi‐frequency bioelectrical impedance analysis. A Cox proportional hazards regression model was used to identify associations of sarcopenia and the combinations of its components with all‐cause mortality and incident disability.

**Results:**

The prevalence of sarcopenia was 11.5% (105/917) in men and 16.7% (156/934) in women. Significant sarcopenia‐related factors other than ageing were hypoalbuminaemia, cognitive impairment, low activity, and recent hospitalization (all *P*‐values <0.05) among men and cognitive impairment (*P* = 0.004) and depressed mood (*P* < 0.001) among women. Individuals with sarcopenia had higher risks of mortality [hazard ratios (95% confidence interval): 2.0 (1.2–3.5) in men and 2.3 (1.1–4.9) in women] and incident disability [1.6 (1.0–2.7) in men and 1.7 (1.1–2.7) in women]. Compared with the individuals without any sarcopenia components, those having low grip strength and/or slow gait speed without low ALMI tended to have an increased risk of disability [1.4 (1.0–2.0), *P* = 0.087], but not mortality [1.3 (0.8–2.2)]. We did not find increased risks of these outcomes in participants having low ALMI in the absence of low grip strength and slow gait speed [1.2 (0.8–1.9) for mortality and 0.9 (0.6–1.3) for incident disability].

**Conclusions:**

Japanese older men and women meeting Asian criteria of sarcopenia had increased risks of all‐cause mortality and disability. There were no significant increased risks of death or incident disability for both participants with muscle weakness and/or low performance without low muscle mass and those with low muscle mass with neither muscle weakness nor low performance. Further studies are needed to examine the interaction between muscle loss, muscle weakness, and low performance for adverse health‐related outcomes.

## Introduction

Sarcopenia is a progressive skeletal muscle disorder that is associated with an increased risk of adverse outcomes including frailty, physical disability, and mortality. Several meta‐analyses of observational studies reported approximately two‐fold to four‐fold higher risk of mortality and functional disability irrespective of differences in the measurement and criteria of sarcopenia.^1^, ^2^, ^3^ According to these meta‐analyses, most previous studies on the relative risks of death and disability in community‐dwelling older adults with sarcopenia have been conducted in Europe and the USA. There have been only a few studies^4^, ^5^, ^6^ with Asian populations, whose body composition, muscle strength, and physical performance are different from those of Western populations. Furthermore, there is limited evidence on sex differences in the effects of sarcopenia on mortality or disability, even in Western countries.^7^

Regarding sarcopenia components, although the specific associations of muscle mass, muscle strength, and physical performance with adverse health‐related outcomes have been documented,^8^, ^9^, ^10^, ^11^, ^12^, ^13^ the effect of their combinations on clinical consequences has not been well examined. In 2018, the European Working Group on Sarcopenia in Older People (EWGSOP) updated the original definition to reflect growing evidence regarding this condition over the last decade,^14^ followed by revised sarcopenia criteria submitted by the Asian Working Group for Sarcopenia in 2019 (AWGS 2019).^15^ The algorithms of sarcopenia by EWGSOP2 and AWGS 2019 included low muscle mass as an essential factor in defining sarcopenia, and thus, individuals who do not have low muscle mass are categorized as non‐sarcopenia, regardless of muscle weakness or low performance. However, the recent definition of sarcopenia by the Sarcopenia Definition and Outcomes Consortium has included both muscle weakness and slowness, but not low lean mass.^16^ Therefore, the impact of the association of lower levels of muscle strength or physical performance with and without low muscle mass on the risk of adverse health‐related outcomes should be further examined to develop an evidence‐based definition of sarcopenia.

The aim of the present study was to investigate the prevalence, associated factors, and magnitude of association with mortality and incident disability for sarcopenia and the combinations of its components among Japanese community‐dwelling older men and women.

## Methods

### Study cohort

The study cohort comprised residents aged 65 years or older who participated in annual health check‐ups between 2008 and 2016 in Kusatsu, a Japanese town in Gunma Prefecture,^17^ and biennial health check‐ups between 2010 and 2014 in Hatoyama, a town in Saitama Prefecture^18^ in Japan. Baseline data for this study were on 1950 participants (1254 in Kusatsu and 696 in Hatoyama) who completed an assessment for sarcopenia at an initial check‐up during the study period. Participants who had missing data were excluded (*n* = 99); consequently, this study included 1851 participants (50.5% women; mean age 72.0 ± 5.9). Written informed consent was obtained from all participants after they were provided with a detailed explanation of the study protocol, which was developed in accordance with the guidelines proposed in the Declaration of Helsinki and was approved by the Ethics Committee of the Tokyo Metropolitan Institute of Gerontology.

### Definition of sarcopenia

Sarcopenia was assessed based on the AWGS 2019 criteria, which are composed of three components: appendicular lean mass, grip strength, and physical function measures.^16^ Appendicular lean mass was measured using direct segmental multi‐frequency bioelectrical impedance analysis (BIA) (InBody 720 analyser, InBody Co., Ltd., Seoul, Korea).^19^ We calculated appendicular lean soft tissue mass (LSTM) from the estimate of LSTM of arms and legs; then, the appendicular lean mass index (ALMI) was calculated by dividing appendicular LSTM by height in m^2^. As evaluated by BIA, the ALMI cut‐off point for sarcopenia was <7.0 kg/m^2^ for men and <5.7 kg/m^2^ for women. We also determined body mass index (BMI), fat‐free mass index (FFMI), and fat mass index (FMI). Handgrip strength (kg) was measured twice in the dominant hand, with the participant squeezing a Smedley‐type handgrip dynamometer (Yagami Co, Tokyo, Japan) as hard as possible; the higher of the two measurements was used in the analysis. The definitions of low grip strength for men and women were <28 and <18 kg, respectively. Gait speed was measured over a straight 11 m walkway marked with tape at 3 and 8 m. Well‐trained observers, using a stopwatch, gauged the time required to walk 5 m at a natural speed and calculated usual gait speed (m/s). The lower level of physical function was evaluated by usual gait speed and defined as <1.0 m/s for both sexes. According to the procedure proposed by the AWGS 2019,^15^ sarcopenia was defined as occurring when participants had low ALMI and low grip strength and/or slow gait speed. The presence of all three of these components was defined as severe sarcopenia. We defined pre‐sarcopenia as occurring when individuals have low ALMI and neither low grip strength nor slow gait speed, or those having low grip strength and/or slow gait speed without low ALMI.

### Measurement of baseline variables

The measurement of other variables at baseline has previously been described in detail.^20^ Risk characteristics included age, BMI, FFMI, ALMI, FMI, handgrip strength, usual gait speed, systolic and diastolic blood pressures, hypertension (systolic blood pressure ≥ 140 mmHg, diastolic blood pressure ≥90 mmHg, or antihypertensive medication use), diabetes mellitus (haemoglobin A1c ≥6.5% or current use of anti‐diabetic medication), anaemia (haemoglobin <13.0 g/dL in men and <12.0 g/dL in women), hypoalbuminaemia (serum albumin ≤3.8 g/dL), illness history of stroke, heart diseases, joint disease, cancer, a history of hospitalization within the past year, current smoking, cognitive impairment (the Mini‐Mental State Examination score ≤23^21^), depressed mood (the short form of the Geriatric Depression Scale ≥5^22^), low activity (answer of ‘less than once a day’ to the question ‘How often do you usually go outdoors?’), and fair or poor self‐rated health.^23^

### Follow‐up and ascertainment of death and disability

We ascertained the occurrence of death and/or disability in Kusatsu as of 13 December 2017 and in Hatoyama as of 31 December 2015. All‐cause mortality was confirmed by checking local registries linking with Japanese National Vital Statistics. Disability was determined to be present when individuals were certified as needing care due to physical or cognitive disability by the Japanese long‐term care insurance (LTCI) system,^24^ which is a mandatory system for every Japanese person aged 40 years and older that provides various types of formal care and support to eligible older adults with disabilities. The certification processes include the assessments of functional disability and cognitive disability and a reference letter from attending local physicians. In this study, the onset of disability was defined as new certification by the LTCI service, using the date of LTCI application as the incident date of disability.

### Statistical analysis

We used univariate analysis of variance to test for differences in age‐adjusted and study area‐adjusted mean values and proportions of baseline characteristics stratified by the presence of sarcopenia. To compute odds ratios and 95% confidence intervals (CIs) of sarcopenia for associated factors, we used multivariate logistic regression analysis. We examined the interactions between sex and each factor by entering interaction terms as the independent variable.

Using Cox proportional hazards regression model, the hazard ratios (HRs) and 95% CIs of the occurrences of all‐cause mortality and incident disability for sarcopenia and pre‐sarcopenia were calculated compared with the reference group consisting of individuals without low ALMI, low grip strength, or slow gait speed. The HRs of incident disability were examined for 1787 participants after excluding those with pre‐existing disabilities (*n* = 64) at baseline. We also calculated the HRs of these outcomes for the subcategories of sarcopenia and pre‐sarcopenia: sarcopenia was subclassified into severe and non‐severe sarcopenia. Pre‐sarcopenic individuals were subclassified as those with low ALMI with neither low grip strength nor slow gait speed and those with low grip strength and/or slow gait speed without low ALMI.

Person‐years were calculated as the sum of individual follow‐up durations until the occurrence of death or disability, or emigration from the community, whichever occurred first. During a mean follow‐up of 5.8 years (maximum, 9.5 years), we identified 178 deaths, 256 incident disabilities, and 339 deaths or disabilities, including 95 persons who died after the disability occurred. We adjusted for age and study area in the initial model calculating HRs stratified by sex and further adjusted for other potential confounding variables, namely, FMI, diabetes, history of stroke, anaemia, hypoalbuminaemia, current smoking, cognitive impairment, depressed mood, and hospitalization, within the past year in the multivariable‐adjusted model among men and women. Probability values for statistical tests were two tailed. *P* < 0.05 was regarded as statistically significant. All analyses were performed with IBM SPSS Statistics Version 23.0 (IBM Corporation, Armonk, NY, USA).

## Results

The total prevalence of sarcopenia was 11.5% in men and 16.7% in women (*Table* 1). Prevalence of sarcopenia increased with age in both men and women, with approximately 22% of men and women aged 75–79 years old and 32.4% of men and 47.7% of women aged 80 years and older.

**Table 1:** Prevalence of sarcopenia

	Men				Women	
	*N*	No. of cases	Prevalence (%)	*N*	No. of cases	Prevalence (%)
65–69 years	379	14	3.7	409	27	6.6
70–74 years	258	19	7.4	229	29	12.7
75–79 years	172	37	21.5	166	38	22.9
80 years and over	108	35	32.4	130	62	47.7
Total	917	105	11.5	934	156	16.7

*Table* 2 shows the characteristics of the participants at baseline according to sarcopenia status. In both men and women, sarcopenic individuals were significantly older than those without sarcopenia and had lower BMI, FFMI, ALMI, FMI, handgrip strength, and usual gait speed after the adjustment for age and study area. Men having sarcopenia had lower systolic and diastolic blood pressures and higher prevalence of current smoking, anaemia, hypoalbuminaemia, cognitive impairment, low activity, fair or poor self‐rated health, and hospitalization within the past year, while women with sarcopenia had lower systolic blood pressure and higher prevalence of cognitive impairment, depressed mood, and fair or poor self‐rated health. According to multiple logistic regression analysis (*Table* 3), the factors that were significantly associated with sarcopenia were age and cognitive impairment in both men and women, as well as hypoalbuminaemia, low activity, and hospitalization within the past year in men and depressed mood in women. There were no significant interactions between sex and each associated factor for the presence of sarcopenia.

**Table 2:** Characteristics of participants according to sarcopenia status

	Men				Women	
	Sarcopenia	No sarcopenia	Age, study area‐adjusted difference (*P*‐value)	Sarcopenia	No sarcopenia	Age, study area‐adjusted difference (*P*‐value)
*n*	105	812		156	778	
Age (years)	77.3 ± 6.3	71.3 ± 5.3	<0.001	77.0 ± 6.9	71.0 ± 5.4	<0.001
BMI (kg/m^2^)	21.4 ± 2.6	23.7 ± 3.0	<0.001	21.4 ± 2.8	23.3 ± 3.3	<0.001
FFMI (kg/m^2^)	15.8 ± 1.2	17.8 ± 1.4	<0.001	14.4 ± 0.9	15.7 ± 1.2	<0.001
ALMI (kg/m^2^)	6.3 ± 0.6	7.4 ± 0.7	<0.001	5.1 ± 0.4	6.1 ± 0.6	<0.001
FMI (kg/m^2^)	5.5 ± 2.2	5.9 ± 2.1	0.004	7.0 ± 2.5	7.6 ± 2.7	0.001
Handgrip strength (kg)	24.4 ± 4.7	35.4 ± 5.7	<0.001	14.6 ± 3.6	22.2 ± 4.0	<0.001
Usual gait speed (m/s)	1.1 ± 0.3	1.4 ± 0.2	<0.001	1.1 ± 0.3	1.4 ± 0.2	<0.001
Systolic blood pressure (mmHg)	132.5 ± 23.1	138.3 ± 21.6	0.014	135.2 ± 21.9	136.1 ± 21.9	0.040
Diastolic blood pressure (mmHg)	75.3 ± 12.4	80.3 ± 12.0	0.046	74.9 ± 12.7	77.5 ± 11.8	0.061
Hypertension (%)	59.0	66.4	0.062	64.7	60.4	n.s.
Diabetes mellitus (%)	20.0	18.1	n.s.	7.7	11.4	0.077
Current smoking (%)	24.8	20.9	0.010	9.0	9.1	n.s.
History of stroke (%)	9.5	7.1	n.s.	5.1	4.5	n.s.
History of heart diseases (%)	20.0	17.9	n.s.	10.3	10.5	n.s.
History of bone and joint disease (%)	19.1	18.5	n.s.	33.3	27.6	n.s.
History of cancer (%)	9.7	11.0	n.s.	7.7	7.7	n.s.
Anaemia (%)	26.7	14.4	0.044	22.4	11.2	0.066
Hypoalbuminaemia (%)	20.0	5.4	<0.001	9.0	4.4	n.s.
Cognitive impairment (%)	14.3	4.2	0.005	13.5	3.4	<0.001
Depressed mood (%)	29.5	20.5	0.086	42.5	22.4	<0.001
Low activity (%)	26.7	12.7	<0.001	29.5	19.4	n.s.
Fair or poor self‐rated health (%)	29.5	14.8	0.001	25.0	12.7	0.001
Hospitalization within the past year (%)	18.1	9.0	0.011	9.6	7.2	n.s.

ALMI, appendicular lean mass index; BMI, body mass index; FFMI, fat‐free mass index; FMI, fat mass index; n.s., not significant (*P* ≥ 0.1).

Values are mean ± standard deviation or percentage. The number of participants with missing data for history of bone and joint diseases, cognitive impairment, and depressed mood was 92, 115, and 27, respectively.

**Table 3:** Multivariate odds ratios of sarcopenia associated with risk factors

	Men	Women	Total	Interaction with sex (*P*‐value)
Sex (men = 1; women = 2)			1.9 (1.4–2.7)***	—
Age (+1 year)	1.2 (1.1–1.2)***	1.2 (1.1–1.2)***	1.2 (1.1–1.2)***	0.594
Current smoking (no = 0; yes = 1)	1.8 (1.0–3.1)*	1.3 (0.7–2.6)	1.4 (0.9–2.2)^+^	0.464
Anaemia (no = 0; yes = 1)	1.3 (0.7–2.3)	1.5 (0.9–2.5)	1.2 (0.8–1.8)	0.986
Hypoalbuminaemia (no = 0; yes = 1)	2.3 (1.2–4.5)*	0.9 (0.4–2.0)	1.5 (0.9–2.5)	0.132
Cognitive impairment (no = 0; yes = 1)	2.3 (1.0–5.5)^+^	2.8 (1.4–5.8)**	2.6 (1.5–4.4)**	0.607
Depressed mood (no = 0; yes = 1)	1.2 (0.7–2.1)	2.4 (1.6–3.6)***	1.9 (1.4–2.6)***	0.094
Low activity (no = 0; yes = 1)	1.8 (1.0–3.3)*	1.2 (0.7–1.8)	1.4 (1.0–2.0)*	0.225
Hospitalization within the past year (no = 0; yes = 1)	2.3 (1.2–4.3)*	1.3 (0.7–2.7)	1.7 (1.1–2.7)*	0.320

Values are odds ratios (95% confidence interval). We adjusted the study area and fat mass index as well.

+ *P* < 0.1.

* *P* < 0.05.

** *P* < 0.01.

*** *P* < 0.001.

Compared with the reference group that did not belong to a low category on any parameters (ALMI, grip strength, or gait speed), the risk for all‐cause mortality and incident disability adjusted for age and study area was approximately three‐fold and two‐fold higher, respectively, in the presence of sarcopenia in both men and women (*Table* 4). The multivariable HRs (95% CI) of mortality and incident disability for sarcopenia were 2.0 (1.2–3.5) and 1.6 (1.0–2.7), respectively, in men and 2.3 (1.1–4.9) and 1.7 (1.1–2.7), respectively, in women. Pre‐sarcopenia was not associated with significantly increased risks of mortality and incident disability in both men and women.

**Table 4:** Incidence and hazard ratios of mortality and incident disability for sarcopenia

	No. at risk	All‐cause mortality			Incident disability			Mortality or incident disability		
		Crude incidence, per 1000 person‐years	Age and study area‐adjusted HR (95% CI)	Multivariable HR (95% CI)	Crude incidence, per 1000 person‐years	Age and study area‐adjusted HR (95% CI)	Multivariable HR (95% CI)	Crude incidence, per 1000 person‐years	Age and study area‐adjusted HR (95% CI)	Multivariable HR (95% CI)
Men										
Reference	540	14.2	1.0	1.0	16.8	1.0	1.0	24.8	1.0	1.0
Pre‐sarcopenia	272	22.5	1.2 (0.8–1.9)	1.0 (0.7–1.7)	29.8	1.0 (0.6–1.5)	0.9 (0.6–1.4)	44.0	1.1 (0.8–1.6)	1.0 (0.7–1.5)
Sarcopenia	105	74.4	3.0 (1.8–5.0)***	2.0 (1.2–3.5)**	85.1	2.0 (1.3–3.3)**	1.6 (1.0–2.7)^+^	116.1	2.3 (1.5–3.4)***	1.9 (1.2–2.9)**
Women										
Reference	483	4.8	1.0	1.0	16.4	1.0	1.0	19.7	1.0	1.0
Pre‐sarcopenia	295	11.6	1.9 (0.9–3.8)	1.9 (0.9–3.8)	25.9	1.2 (0.8–1.9)	1.2 (0.8–1.9)	32.3	1.3 (0.9–1.9)	1.3 (0.9–1.9)
Sarcopenia	156	28.2	2.9 (1.4–5.8)**	2.3 (1.1–4.9)*	69.5	1.9 (1.2–2.9)**	1.7 (1.1–2.7)*	82.8	2.1 (1.4–3.1)***	1.9 (1.3–3.0)**

CI, confidence interval; HR, hazard ratio.

Reference was a group of the participants without low appendicular lean mass index (ALMI), low grip strength, or slow gait speed. Pre‐sarcopenia was composed of participants who had low ALMI with neither low grip strength nor slow gait speed and those who had low grip strength and/or slow gait speed without low ALMI. Multivariable HR was adjusted for age, study area, fat mass index, diabetes, history of stroke, anaemia, hypoalbuminaemia, current smoking, cognitive impairment, depressed mood, and hospitalization for the past year.

+ *P* < 0.1.

* *P* < 0.05.

** *P* < 0.01.

*** *P* < 0.001.

*Table* 5 shows the multivariable HRs of mortality and incident disability for the subcategories of pre‐sarcopenia and sarcopenia among men and women. Compared with individuals without any sarcopenia components, those having low grip strength and/or slow gait speed without low ALMI were associated with increased risks of incident disability, and mortality or incident disability with marginal significance [HRs (95% CI): 1.4 (1.0–2.0), *P* = 0.087, and 1.4 (1.0–1.9), *P* = 0.057, respectively], but not all‐cause mortality [1.3 (0.8–2.2), *P* = 0.346]. The other subcategory of pre‐sarcopenia, low ALMI with neither low handgrip strength nor slow gait, did not show increased risk of each outcome. The HRs of death and incident disability for severe sarcopenia were about three‐fold higher, while those for non‐severe sarcopenia were less than double.

**Table 5:** Multivariable hazard ratios of mortality and incident disability for the subcategories of pre‐sarcopenia and sarcopenia among men and women

	All‐cause mortality		Incident disability		Mortality or incident disability	
	Crude incidence, per 1000 person‐years	Multivariable HR (95% CI)	Crude incidence, per 1000 person‐years	Multivariable HR (95% CI)	Crude incidence, per 1000 person‐years	Multivariable HR (95% CI)
Reference (*n* = 1023)	9.5	1.0	16.6	1.0	22.3	1.0
Pre‐sarcopenia						
Low grip strength and/or slow gait speed without low ALMI (*n* = 190)	18.9	1.3 (0.8–2.2)	48.3	1.4 (1.0–2.0)^+^	57.8	1.4 (1.0–1.9)^+^
Low ALMI with neither low grip strength nor slow gait speed (*n* = 377)	15.5	1.2 (0.8–1.9)	18.2	0.9 (0.6–1.3)	28.5	1.0 (0.7–1.4)
Sarcopenia						
Non‐severe sarcopenia (*n* = 201)	36.4	1.8 (1.2–2.9)**	59.2	1.6 (1.1–2.3)*	77.2	1.7 (1.2–2.4)**
Severe sarcopenia (*n* = 60)	74.7	3.2 (1.8–5.7)***	203.7	3.0 (1.8–5.1)***	237.7	3.3 (2.1–5.3)***

ALMI, appendicular lean mass index; CI, confidence interval; HR, hazard ratio.

Reference was a group of the participants without low ALMI, low grip strength, or slow gait speed. Multivariable HR was adjusted for age, sex, study area, fat mass index, diabetes, history of stroke, anaemia, hypoalbuminaemia, current smoking, cognitive impairment, depressed mood, and hospitalization for the past year.

+ *P* < 0.1.

* *P* < 0.05.

** *P* < 0.01.

*** *P* < 0.001.

## Discussion

We found that approximately one‐fifth of both men and women aged 75–79 years and about one‐third of men and about one‐half of women aged 80 years and older had sarcopenia as defined by AWGS 2019. Although it is difficult to compare the prevalence in this cohort with those in previous studies of other Japanese community‐dwelling populations^25^, ^26^, ^27^ due to differences in the population representativeness and the definition, including cut‐off values of sarcopenia, sarcopenia is highly prevalent among older adults after 75 years of age, as evident across the population‐based studies conducted in Japan as well as in other countries.^28^, ^29^

In our study, we found sex‐specific patterns of correlates with sarcopenia. According to multivariate data analysis, significant sarcopenia‐related factors in addition to ageing were hypoalbuminaemia, cognitive impairment, low activity, and recent hospitalization among men and cognitive impairment and depressed mood among women. Previous studies have shown that hypoalbuminaemia,^30^, ^31^, ^32^ depressive symptoms,^33^, ^34^ and cognitive decline^35^, ^36^ were factors associated with sarcopenia, muscle loss, muscle weakness, and slow gait speed, which support our findings. In consideration of the insignificant interaction of sex and each associated factor for sarcopenia, the sex differences in the correlates with sarcopenia found in the current study may be due to the differences in the prevalence of each factor: men had a higher prevalence of hypoalbuminaemia and hospitalization within the past year, while depressed mood was more common among women in our cohort. History of hospitalization within the previous year and low activity (low frequency of outings) may be linked to physical inactivity that causes a decline in muscle strength.^37^ Considering the reason that a low frequency of outings was not a significant factor for sarcopenia among women in this study, we infer that many older Japanese women have experienced increased physical activity through household activities.^38^

Notably, our 5.8 year follow‐up study of 1851 Japanese community‐dwelling older adults showed that sarcopenic men and women had about two‐fold higher risks of all‐cause mortality and occurrence of disability compared with the reference group after the adjustment for the potential confounders. Severe sarcopenia increased the risks of death and incident disability by about three times. The risk ratios for sarcopenia in our study were similar to those in previous meta‐analyses of observational studies that reported approximately two‐fold to four‐fold higher risk of mortality and functional disability,^1^, ^2^, ^3^ beyond the differences in the populations and measurement, cut‐off values, and criteria of sarcopenia. Regarding the sex differences in the impact of sarcopenia on mortality, two Japanese and Korean studies with fewer than 1000 participants reported that an elevated risk of all‐cause mortality for sarcopenia was only observed in men.^4^, ^5^ In contrast, two community‐based studies in Hong Kong and the USA with over 4000 participants showed a higher risk of death due to sarcopenia for both men and women,^6^, ^7^ which was similar to our findings.

The current study demonstrated interesting findings regarding the effect of pre‐sarcopenia and its subcategories on adverse health‐related outcomes. Pre‐sarcopenia, which does not belong to either the reference group or the sarcopenia group, was not associated with significantly increased risks of mortality and incident disability. As part of the results, we found that low ALMI in the absence of low grip strength and slow gait speed did not significantly increase the risks of mortality and incident disability. In agreement with our findings, the Health, Aging, and Body Composition study reported that low muscle area did not continue to be a significant factor associated with incident mobility limitations after adjustment for muscle strength among well‐functioning older adults.^39^ The InCHIANTI Study indicated that low lean mass alone is a poor predictor of death and disability and that lower muscle strength is a critical predictor for both physical disability and mortality among community‐dwelling older adults.^40^ Our findings supported the validity of current major algorithms of sarcopenia by EWGSOP2^14^ and AWGS 2019,^15^ which requires both low muscle mass and low muscle strength and/or slow gait speed for sarcopenia definition.

Low grip strength and/or slow gait speed without low ALM tended to be associated with an increased risk of disability. The present finding was compatible with the reporting in the InCHIANTI Study,^41^ which showed that gait speed was the strongest predictor of incident activities of daily living disability, while grip strength was not a statistically significant predictor of incident disability after adjustment for potential confounders including fat‐adjusted lean mass and muscle density. Meanwhile, our finding that low grip strength and/or slow gait speed in the absence of low ALMI did not increase risk of all‐cause mortality may cause controversy when compared with those of previous studies. We examined the dose relationship of grip strength and gait speed with mortality risk stratified by the presence of low ALMI, using fractional polynomial functions (data not shown). Consequently, men without low ALMI had no significant effect of grip strength on mortality risk, whereas women without low ALMI had a significant inverse dose–response relationship between grip strength and mortality. In addition, usual gait speed had no significant dose–response relationship with mortality risk in men and women without low ALMI. We interpreted that these insignificant associations between physical function and mortality risk made the association of low grip strength and/or slow gait speed in the absence of low ALMI with mortality less significant. According to the Health ABC study, the inverse relationship between grip strength and mortality was only slightly attenuated after adjustment for leg muscle area or lean mass.^42^ In a follow‐up study of British older adults by the UK Department of Health and Social Security, neither muscle area nor fat‐free mass explained the increased mortality associated with poorer grip strength in men.^43^ However, this British study found no statistically significant associations between grip strength and all‐cause mortality after adjustment for fat‐free mass in women, suggesting that the effect of lower grip strength on mortality may be attributed partly to low muscle size. There may be several inferences about the suggestive mechanism that the risk of mortality was not significantly high in participants without low muscle mass, despite having low grip strength or slow gait speed. The first mechanism to consider is a muscle's beneficial property, namely, that muscle plays a major role in glucose and lipid metabolism, as well as being a storage organ for free amino acids that substantially increases in demand when malnutrition occurs. As mentioned earlier, there was no significant dose–response relationship between grip strength and mortality in the absence of low ALMI in men who have more muscle mass than women in the current study; therefore, the positive effects of muscles may appear stronger in men. Second, we inferred that participants with low muscle strength or slow gait speed comprised both those who have had naturally low strength or slow speed irrespective of muscle mass and those who were getting lower or slower with age through the process of dynapenia.^44^ While the latter group has a higher risk of death, the former may not. The Baltimore Longitudinal Study of Aging supported this inference: among men under 60 years old, a significant predictor of all‐cause mortality was a decline in grip strength, but not grip strength at baseline.^45^ Additionally, according to our previous follow‐up study of older adults in Kusatsu town, age‐related declining trends in trajectory patterns of grip strength and gait speed were stronger predictors for all‐cause mortality than those measured values at baseline.^46^

Our study has several limitations. First, the study participants were limited to individuals who had undergone health check‐ups in two communities. The participation rates to census population were 55% in Kusatsu, 16% in Hatoyama, and 30% in the two communities combined. Therefore, the prevalence of sarcopenia in the present study does not represent the general population and may be underestimated because of healthy subject bias. Second, the small numbers of outcomes prevented examining the sex‐specific effect for the subcategories of pre‐sarcopenia and the impact of low grip strength and slow gait speed separately in combination with the ALMI category on mortality and incident disability. Third, most of our study participants who had a history of cancer have been completely cured of their cancer; hence, the association of a history of cancer with sarcopenia with mortality in the current study is probably underestimated. Fourth, compared with dual X‐ray absorptiometry, the BIA used in this study likely underestimates appendicular lean mass, although we confirmed that direct segmental multi‐frequency BIA had acceptable accuracy.^19^, ^47^ Fifth, we could not examine the association of sarcopenia with cause‐specific mortality due to the lack of causes of death, which is a subject for future studies. Finally, although we demonstrated associations of sarcopenia and pre‐sarcopenia with mortality and incident disability, futures studies are needed to evaluate their predictive ability related to these outcomes.

Despite these limitations, the present findings suggest that preventing and/or improving sarcopenia may be beneficial in extending healthy life expectancy for community‐dwelling older adults. With respect to the clinical and public health implications of our findings, an assessment of sarcopenia should be introduced to health check‐ups and clinical evaluations of older adults.

In conclusion, this long‐term prospective study involving older Japanese residents demonstrated the prevalence, associated factors, and increased risks of all‐cause mortality and disability for sarcopenia. Muscle weakness or low performance without low muscle mass and low muscle mass with neither muscle weakness nor low performance were not significantly associated with an increased risk of death or incident disability. Further studies are needed to examine the interaction between muscle loss, muscle weakness, and low performance affecting adverse health‐related outcomes.

## Funding

This work was supported by Grants‐in‐Aid for Scientific Research (B) (Grant Numbers JP20390190, JP21390212, JP24390173, JP26310111, and 17H04140) from the Ministry of Education, Culture, Sports, Science and Technology, Japan, and Research Funding for Longevity Sciences from the National Center for Geriatrics and Gerontology (Grant Number 29‐42), Japan.

## Author contributions

A.K. conceived and designed the study, analysed and interpreted the data, and drafted the manuscript. S. Seino, T.A., Y.N., Y.Y., H.A., M.N., Y.T., M.N., Y.F., and S. Shinkai acquired the data and critically revised the manuscript for key intellectual content. S. Shinkai also supervised the study. A.K. and S. Seino are the guarantors of this work and, as such, had full access to all the data in the study and take responsibility for the integrity of the data and the accuracy of the data analysis. All authors approved the final version of the manuscript.

## Conflict of interest

None declared.
